# Quality of life and functional outcomes after laparoscopic total mesorectal excision (LaTME) and transanal total mesorectal excision (taTME) for rectal cancer. an updated meta-analysis

**DOI:** 10.1007/s00384-024-04703-x

**Published:** 2024-08-09

**Authors:** Sara Lauricella, Francesco Brucchi, Francesco Maria Carrano, Diletta Cassini, Roberto Cirocchi, Patricia Sylla

**Affiliations:** 1 Colorectal Surgery Division, Department of Surgery, Fondazione IRCCS Istituto Nazionale Dei Tumori, 20133 Milan, Italy; 2 University of Milan, 20122 Milan, Italy; 3 Department of Medical and Surgical Sciences and Translational Medicine, Faculty of Medicine and Psychology, St Andrea Hospital, Sapienza University, Rome, Italy; 4 ASST Nord Milano-Department of General Surgery, Sesto San Giovanni Hospital, Sesto San Giovanni, MI Italy; 5 Digestive and Emergency Surgery Unit, S.Maria Hospital Trust, 05100 Terni, Italy; 6 Division of Colon and Rectal Surgery, Mount Sinai Health System, New York, NY USA

**Keywords:** Transanal TME, Laparoscopic TME, Functional outcomes, Quality of Life, Rectal cancer, Meta-analysis

## Abstract

**Purpose:**

Concerns exist regarding the potential for transanal total mesorectal excision (TaTME) to yield poorer functional outcomes compared to laparoscopic TME (LaTME). The aim of this study is to assess the functional outcomes following taTME and LaTME, focusing on bowel, anorectal, and urogenital disorders and their impact on the patient’s QoL.

**Methods:**

A systematic review was performed according to the Preferred Reporting Items for Systematic Reviews and Meta-Analyses (PRISMA) guidelines and A Measurement Tool to Assess systematic Reviews (AMSTAR) guidelines. A comprehensive search was conducted in Medline, Embase, Scopus, and Cochrane Library databases. The variables considered are: Low Anterior Resection Syndrome (LARS), International Prostate Symptom Score (IPSS) and Jorge-Wexner scales; European Organisation for Research and Treatment of Cancer (EORTC) QLQ-C29 and QLQ-C30 scales.

**Results:**

Eleven studies involving 1020 patients (497-taTME group/ 523-LaTME group) were included. There was no significant difference between the treatments in terms of anorectal function: LARS (MD: 2.81, 95% CI: − 2.45–8.08, *p* = 0.3; I2 = 97%); Jorge-Wexner scale (MD: -1.3, 95% CI: -3.22–0.62, p = 0.19). EORTC QLQ C30/29 scores were similar between the groups. No significant differences were reported in terms of urogenital function: IPSS (MD: 0.0, 95% CI: − 1.49–1.49, *p* = 0.99; I^2^ = 72%).

**Conclusions:**

This review supports previous findings indicating that functional outcomes and QoL are similar for rectal cancer patients who underwent taTME or LaTME. Further research is needed to confirm these findings and understand the long-term impact of the functional sequelae of these surgical approaches.

**Supplementary Information:**

The online version contains supplementary material available at 10.1007/s00384-024-04703-x.

## Introduction

Total mesorectal excision (TME) is the gold standard treatment for middle and lower rectal tumors. The technique involves the complete, en bloc removal of the mesorectal fat with associated lymph nodes, which is pivotal for achieving low local recurrence rates [1].

The evolution of TME surgery from open to laparoscopic, robotic, and transanal approaches, accompanied by significant technological advancements, has improved surgical outcomes and minimized invasiveness.

Transanal TME (taTME) is the latest advancement, pioneered to tackle insidious pelvic dissections required for tumors located in the lower third of the rectum [2]. First described by Sylla et al. in 2010, it has since seen a progressive and wide adoption in clinical practice [3]. taTME offers the advantage of improved visibility and access to the distal rectum, with the aim of achieving a more precise dissection that may lead to a lower rate of positive circumferential resection margins and better preservation of autonomic nerves. This approach appears particularly advantageous in case of patients with anatomic constraints that make LaTME challenging, including a narrow pelvis, obesity, and low-lying tumors [2, 4–8]. Several studies indicate that taTME may offer advantages over LaTME, including a lower conversion rate to open surgery, wider circumferential resection margins (CRM), and lower rates of positive CRM involvement. [9–12] However, in terms of oncological outcomes, when performed in high-volume centers, both taTME and LaTME achieve equivalent resection quality and show similar local recurrence rates [9–11, 13, 14]. Perioperative outcomes such as estimated blood loss, hospital stay, intraoperative complications, and postoperative complications do not show significant differences between the two approaches [9–12, 15–17]. However, in some studies, taTME has been associated with shorter operative times, lower overall morbidity, and reduced rates of anastomotic leak compared to LaTME [10, 12, 17–19]. Conversely, there have been increased concerns about reports of higher incidence of postoperative fecal incontinence following taTME. [13]

While the long-term outcomes and comparisons with standard laparoscopic or robotic rectal resections are still being evaluated [20, 21], data about the functional sequelae from both laparoscopic and transanal approaches and their impact on patient’s quality of life (QoL) are still limited [22].

While some variability exists in the literature [23], evidence suggests that taTME might initially be associated with more significant functional impairments, though these differences may diminish over time [24, 25].

This paper aims to assess the comparative functional outcomes following taTME and LaTME, focusing on bowel, anorectal, and urogenital disorders and their impact on the patient’s QoL.

## Material and methods

### Data sources and searches

The peer-reviewed literature published from January 1982 to May 2024 was searched using Medline (PubMed), Embase, Scopus, and Cochrane Library databases with MeSH terms [rectal neoplasm OR cancer] AND [transanal TME OR laparoscopic TME OR “Total Mesorectal Excision”] AND [“function” OR “functional outcomes” OR “Quality of Life”], and with limits “Title/Abstract, Human Subjects, English”.

This meta-analysis was performed in accordance with the Preferred Reporting Items for a Systematic Review and Meta-analysis of Diagnostic Test Accuracy Studies (PRISMA-DTA) Statement, Meta-analyses Of Observational Studies in Epidemiology (MOOSE) guidelines and A Measurement Tool to Assess systematic Reviews (AMSTAR) guidelines [26–28]. The planned protocol of this meta-analysis was registered in PROSPERO (PROSPERO 2023: CRD42024540266). In addition, the reference lists of retrieved articles were screened to identify further studies. The final aim of the search was to identify studies comparing taTME vs LaTME in terms of functional outcomes and Quality of Life in adult patients to provide a synthesis of the scientific evidence by the meta-analysis process.

### Study selection

Two investigators (SL and FB) independently screened titles and abstracts to identify potentially eligible studies using Rayyan systematic review software [29] and confirmed eligibility by reading the full-text publication of selected records. Any discrepancies were resolved by consensus or arbitration by a third reviewer (PS). Studies were considered eligible if they included adult patients diagnosed with rectal cancer, compared transanal total mesorectal excision (taTME) to laparoscopic total mesorectal excision (LaTME), and reported on functional outcomes and quality of life (QoL).

No geographic or language restrictions were applied. Papers were excluded if they reported duplicative results from the same authors’ group, if they lacked sufficient data, or in case of non-comparative studies, reviews, meta-analyses, letters, case reports, or conference abstracts.

### Data extraction and quality assessment

Two authors examined the main features of each retrieved article, reporting the following data: (a) study characteristics: the first author, country, year of publication, number of patients, study type; (b) patient baseline: tumor site, gender, age, body mass index (BMI), American Society of Anesthesiologists (ASA) class, rectal cancer distance from the anal verge, tumor staging, neoadjuvant treatment, protective ileostomy, time of ileostomy reversal, time of follow up from index surgery, and previous functional impairments; (c) study outcomes: (1) functional results: Low Anterior Resection Syndrome (LARS) scale [30, 31], International Prostate Symptom Score (IPSS) [32] and Jorge-Wexner scale [33]; (2) the QoL: European Organisation for Research and Treatment of Cancer (EORTC) QLQ-C29 [34] and QLQ-C30 [35, 36] scales.

### Data synthesis and analysis

Categorical data were collected as absolute numbers. If reported as median and range, these were converted to mean and standard deviation (SD) using the method described by Wan et al. [37] A random-effects model was used for the meta-analysis of all outcomes. All estimates were presented with a 95% confidence interval (CI). A continuity correction of 0.5 was applied in studies with zero cell frequencies to calculate confidence limits and standard errors.

Heterogeneity among effect size (ES) results was assessed using the Q and I^2^ statistics. I^2^ values of 25%, 50%, and 75% represented low, moderate, and high heterogeneity, respectively [38]. All analyses were performed using Review Manager (RevMan, Version 5.4.1). When high heterogeneity was detected, a sensitivity analysis was performed to assess the robustness of the overall findings by systematically excluding individual studies or subgroups to determine their impact on the pooled effect estimates.

### Risk of bias assessment

The quality of non-randomized controlled trials (NRCTs) was assessed using the Newcastle–Ottawa Scale (NOS) [39], with scores ranging from 7 to 8 stars, indicating good quality. Two researchers independently assessed the study using the Review Manager tool, focusing on five key domains: bias arising from the randomization process, deviations from intended interventions, missing outcome data, measurement of the outcome, and selection of the reported result. Discrepancies were resolved through consultation with a third-party expert. The detailed risk of bias assessment is provided in the supplementary materials. In accordance with Cochrane guidelines, publication bias was not assessed as fewer than ten studies were included in each data comparison. [40]

## Results

### Study characteristics

**Fig. 1:**
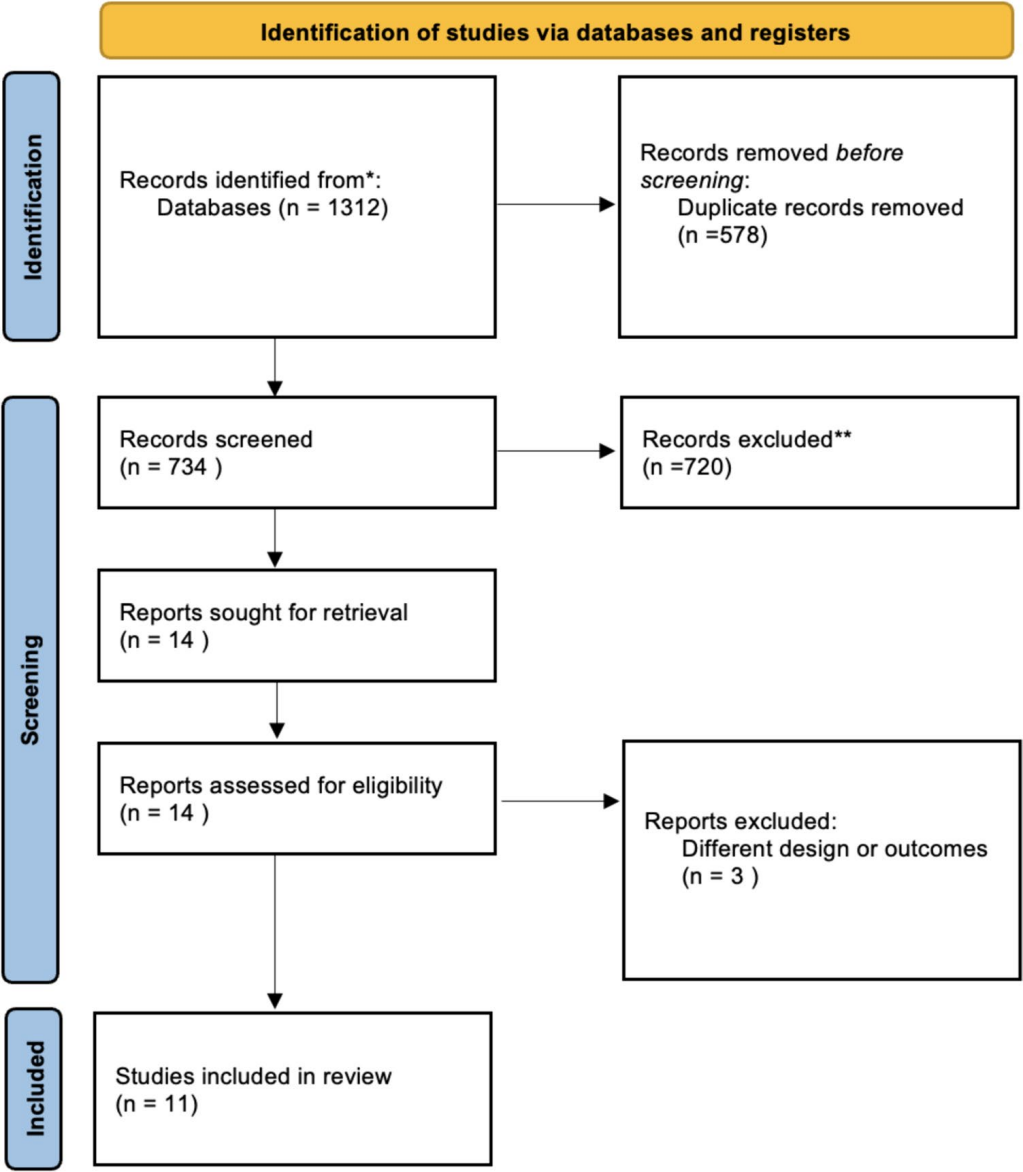
Preferred Reporting Items for Systematic Reviews and Meta-analysis (PRISMA) flowchart of the literature search

The initial literature search retrieved 1312 publications. Of these, 11 studies [19, 21, 41–49] were included in the meta-analysis (Fig. 1), involving 1020 patients (497 in the taTME group and 523 in the LaTME group). Among the included studies, eight were retrospective studies [41–44, 46–49], three prospective cohort studies [19, 45, 50].

### Patient characteristics

The baseline characteristics of patients from the included studies are presented in Table 1 and Table 2, allowing for comparison between the taTME and LaTME groups. Demographic parameters and the use of neoadjuvant treatment were assessed.

**Table 1:** Study characteristics

Author and year	Study period	No. of centres, country	Study design	Functional outcome assessment	No of patients	
					TaTME	LaTME
Seow-En et al. [44], 2024	2021–2022	1, Singapore	Retrospective, PS matched	LARS, Jorge-Wexner scale	12	36
Yang et al. [49], 2023	2019–2021	1, China	Retrospective	LARS, Jorge-Wexner scale	17	34
Li et al. [50], 2021	2014–2018	1, China	Prospective	QLQ-C29, LARS, Jorge-Wexner scale	30	30
Kyong Ha et al. [45], 2021	2014–2017	1, Korea	Prospective, PS matched	LARS, IPSS, QLQ-C30	202	202
Foo et al. [46], 2020	2016–2018	1, China	Retrospective	LARS, Jorge-Wexner scale	35	35
Bjoern et al. [41], 2019	2010–2017	1, Denmark	Retrospective, prosp. DB	LARS, IPSS, EORTC QLQ-C30, EORTC QLQ-C29	49	36
Rubinkiewicz et al. [48], 2019	2013–2017	1, Poland	Retrospective, prosp. DB	LARS, Jorge-Wexner scale	23	23
Dou et al. [43], 2019	2016–2017	1, China	Retrospective	LARS	54	53
Mora et al. [47], 2018	2011–2014	1, Spain	Retrospective, prosp. DB	LARS, EORTC QLQ-C30, EORTC QLQ C-29	16	15
Veltcamp Helbach et al. [42], 2018	2010–2012	1, The Netherlands	Retrospective	LARS, EORTC QLQ-C30, EORTC QLQ C-29, IPSS	27	27
de' Angelis et al. [19], 2015	2011–2014	1, France	case-matched study	Jorge-Wexner scale	32	32

**Table 2:** Patients characteristics

Author and year	Age, mean (SD)		Sex ratio, M/F, (%)		Neoadjuvant CRTx, n (%)			NOS
	TaTME	LaTME	TaTME	LaTME	TaTME	LaTME	p value	7
Seow-En et al. [44], 2024	69.3 ( 6.2)	67.9 ± 11.2	8 (66.7)/4 (33.3)	23 (63.9)/ 13 (36.1)	2 (16.7)	10 (27.8)	0.829	7
Yang et al. [49], 2023	62.88 (10.37)	63.74 ± 11.07	11 (64.71)/ 6 (35.29)	19 (55.88)/ 15 (44.12)	6 (35.29)	16 (47.06)	0.617	8
Li et al. [50], 2021	NR	NR	14 (47)/ 16 (53)	13 (43)/ 17 (57)	17 (57)	15 (50)	0.446	8
Kyong Ha et al. [45], 2021	62.43 ( 9.98)	61.46 ± 11.24	129 (63.9)/ 73 (36.1)	131 (64.9)/ 71 (35.1)	129 (63.9)	118 (58.4)	0.262	7
Foo et al. [46], 2020	67 (25.93)	68 (27.41)	24 (68.6)/11 (31.4)	23 (65.7)/12 (34.3)	14 (40)	15 (42.9)	1.000	8
Bjoern et al. [41], 2019	64.88 (9.645)	62.42 (10.146)	37 (75.5)/12 (24.5)	16 (44.4)/20 (55.6)	8 (16.3)	8 (22.2)	0.492	8
Rubinkiewicz et al. [48], 2019	60 (11.85)	64 (6.67)	13 (56.5)/10 (43.5)	13 (56.5)/10 (43.5)	18 (78.3)	19 (82.6)	0.71	8
Dou et al. [43], 2019	57.5 (37.78)	62 (29.63)	35 (64.8)/19 (35.2)	35 (66)/18 (34)	12 (22.2)	NR	NR	7
Mora et al. [47], 2018	64 (NR)	59.9 (NR)	12 (75)/4 (25)	10 (66.7)/5 (33.3)	7 (43.75)	NR	NR	6
Veltcamp Helbach et al. [42], 2018	68 (5.33)	62.7 (4.52)	18 (66.7)/9 (33.3)	20 (74)/7 (26)	18 (66.67)	22 (81.5)	0.395	7
de' Angelis et al. [19], 2015	64.9 (10.0)	67.2 (9.6)	21 (65.6)/11 (34.4)	21 (65.6)/11 (34.4)	27 (84.4)	23 (71.8)	0.365	8

Author and year	Distance from a.v. (cm)			Tumor staging			Protective ileostomy, n (%)			Time of ileostomy reversal (mo)			Time of follow up from index surgery (mo)		
	taTME	LaTME	p value	taTME	LaTME	p value	taTME	LaTME	p value	taTME	LaTME	p value	taTME	LaTME	p value
Seow-En et al. [44], 2024	NR	NR	NR	pCR = 1 T1 = 2 T2 = 0 T3 = 9 T4 = 0 N0 = 5 N1 = 7 N2 = 0	pCR = 2 T1 = 3 T2 = 9 T3 = 20 T4 = 2 N0 = 25 N1 = 9 N2 = 2	0.035*	NR	NR	NR	5 ± 3			7 ± 6.5	28 ± 14	< 0.05*
Yang et al. [49], 2023	4.03 ± 0.86	4.32 ± 0.75	0.251	I = 4 II = 7 III = 6	I = 4 II = 14 III = 15	0.7	NR	NR	NR	NR	NR		18.56 ± 4.35	17.86 ± 6.36	0.645
Li et al. [50], 2021	< 5 cm = 11 ≥ 5 cm = 19	< 5 cm = 13 ≥ 5 cm = 17	0.778	T0/1 = 12 T2/3/4 = 18	T0/1 = 13 T2/3/4 = 17	0.793	28 (93.3)	29 (96.7)	0.776	7.8 ± 8	8.1 ± 10	0.83	3 and 12	3 and 12	NR
Kyong Ha et al. [45], 2021	≤ 5 cm = 98 ≤ 10 cm = 94 > 10 cm = 10	≤ 5 cm = 83 ≤ 10 cm = 111 > 10 cm = 8	0.238	T1 = 24 T2 = 24 T3 = 136 T4 = 18	T1 = 24 T2 = 27 T3 = 135 T4 = 16	0.96	151 (74.8)	168 (83.2)	0.038	3 months postoperatively or 1 month after adjuvant therapy			12	12	NR
				N- = 61 N + = 141	N- = 66 N + = 136	0.592									
Foo et al. [46], 2020	7 ± 8	7 ± 8	0.953	T1/2 = 26 T3/4 = 9	T1/2 = 17 T3/4 = 17	0.05	27 (77.1)	8 (87.5)	0.347	8 ± 9	8.5 ± 11	0.146	3,6 and 12	3,6 and 12	NR
Bjoern et al. [41], 2019	8.35 ± 1.727	8.14 ± 1.885	0.599	T2 = 25 T3 = 23 T4 = 1	T2 = 17 T3 = 19 T4 = 0	0.625	49 (100)	36 (100)	NR	3 months postoperatively or until the completion of adjuvant CT			22.69 ± 10.308	75.08 ± 17.609	< 0.001*
				N0 = 35 N1 = 6 N2 = 8	N0 = 10 N1 = 10 N2 = 16	< 0.001*									
Rubinkiewicz et al. [48], 2019	3 ± 2	4 ± 2	*0.01	T1 = 2 T2 = 3 T3 = 15 T4 = 3	T1 = 3 T2 = 6 T3 = 12 T4 = 2	0.24	23 (100)	23 (100)	NR	NR		NR	Follow up at 6 months after ileostomy reversal		NR
				N—= 13 N + = 23	N—= 14 N + = 23	0.76									
Dou et al. [43], 2019	< 5 cm = 22 ≥ 5 cm = 32	< 5 cm = 25 ≥ 5 cm = 28	> 0.05	NR	NR	NR	20 (37)	34 (64.2)	NR	NR	NR	NR	NR	NR	NR
Mora et al. [47], 2018	7.44	7.93	0.723	0 = 0 I = 5 II = 7 III = 2	0 = 3 I = 6 II = 3 III = 3	0.143	16 (100)	15 (100)	NR	NR	NR	NR	NR	NR	NR
Veltcamp Helbach et al. [42], 2018	Low = 9 Mid = 14 High = 4	Low = 7 Mid = 18 High = 2	0.569	T0/1 = 4 T2 = 12 T3 = 11	T0/1 = 6 T2 = 9 T3 = 12	0.647	22 (81.5)	22 (81.5)	NR	6 weeks after surgery		NR	20 ± 37.8	59.5 ± 42.3	0.000*
de' Angelis et al. [19], 2015	4 ± 2.5	3.7 ± 2.5	0.631	T2 = 13 T3 = 17 T4 = 2	T2 = 16 T3 = 13 T4 = 3	0.593	32 (100)	32 (100)	NR	NR	NR	NR	32.06 ± 12.1	62.91 ± 12.3	< 0.05*
				N0 = 21 N1 = 10 N2 = 1	N0 = 14 N1 = 15 N2 = 3	0.183									

There were no substantial differences in demographics between groups across studies. The proportion of males was slightly higher in the taTME group (65%) compared to the LaTME group (62%). Mean age was similar between groups, with a negligible difference of -0.541 years (95% CI: -2.951 to 1.869; *p* = 0.66). Based on five studies, the mean BMI difference between groups was 1.18 (95% CI: -59.03 to 61.39; *p* = 0.5). Neoadjuvant chemoradiotherapy was administered to a slightly higher percentage of patients in the taTME group (61.4%) compared to the LaTME group (54.5%), but this difference was not statistically significant (*p* = 0.08). The distance from the anal verge has not shown statistical significance in any studies, with the exception of the study published by Rubinkiewicz et al. (taTME = 3 ± 2; LaTME = 4 ± 2; *p* = 0.01) [48].

Three studies examined previous functional impairments [45, 46, 48].

Kyong Ha et al. [45] revealed that major LARS was found in 19.1% of patients in the taTME group vs 13.6% in the LaTME group. No statistical significant difference was reported. Additionally, no patient experienced fecal incontinence before treatment.

Foo et al. [46] showed that the median preoperative baseline Wexner score was 0 for both groups.

Rubinkiewicz et al. [48] revealed that the median preoperative LARS score were 0 (IQR: 0–5) and 5 (0–21) in LaTME and TaTME groups, respectively (*p* = 0.10). Furthermore, there was no significant difference for the median preoperative Wexner score between groups (*p* = 0.20).

Finally, only four studies [42, 45, 49, 50] have reported their experience with taTME.

A detailed descriptive analysis of functional outcomes and quality of life is provided in Tables 3 and 4 and Tables 1s-2s in the Supplementary materials.

**Table 3:** EORTC QLQ-C29

EORTC QLQ-C29		Li et al. [50] (2021)			Bjoern et al. [41] (2019)			Mora et al. [47] (2018)			Veltcamp Helbach et al. [42] (2018)		
		TaTME	LaTME	p	TaTME	LaTME	p	TaTME	LaTME	p	TaTME	LaTME	p
Functional scales	Body image	81	83.5	0.730	89.34	88.58	0.647	90.97	85.19	0.432	88.40	90.90	0.325
	Anxiety	70	66	0.297	79.59	81.48	0.954	72.92	64.44	0.489	74.40	75.30	0.715
	Weight	72	71	0.836	84.35	86.11	0.605	66.67	77.78	0.361	87.20	84.10	0.493
	Sexual interest (men)	34	36	0.426	50.45 (37)	50 (20)	0.959	53.33 (12)	44.44 (10)	0.629	68.9 (15)	63.3 (20)	0.564
	Sexual interest (women)	25	34.5	0.039*	5.55 (12)	20.83 (16)	0.053	83.33 (4)	88.89 (5)	0.715	83.3 (6)	73.3 (5)	0.662
Symptom scales	Urinary frequency	23	24	0.650	11.90	19.44	0.516	NR	NR	NR	38.90	28.40	0.101
	Blood + mucus stool	1	3	0.102	4.76	0.92	0.183	NR	NR	NR	3.70	3.70	1.000
	Stool frequency	19	19.5	0.860	19.79	17.12	0.440	25.64	36.11	0.327	36.50	30.70	0.556
	Urinary incontinence	15	15.5	0.910	2.04	3.70	0.674	8.33	8.89	0.919	7.40	9.90	0.886
	Dysuria	4.5	4	0.903	2.04	1.85	0.771	4.44	6.67	0.765	2.50	1.20	0.556
	Abdo pain	7.5	13	0.053	8.16	11.11	0.329	11.11	28.89	0.044*	10.30	7.40	0.643
	Buttock pain	9.5	11	0.472	14.28	2.77	0.011*	18.75	28.89	0.335	24.70	12.30	0.114
	Bloating	18	24.5	0.061	17.68	12.96	0.362	14.58	37.78	0.042*	14.80	14.80	1.000
	Dry mouth	19	26	0.087	18.36	10.18	0.387	NR	NR	NR	29.80	8.60	0.156
	Hair loss	12.5	10	0.581	2.72	1.85	0.896	NR	NR	NR	9.90	0.00	0.010*
	Taste	9.1	9	0.821	4.16	0.00	0.047*	NR	NR	NR	17.30	6.20	0.083
	Flatulence	70	66	0.940	32.65	26.85	0.392	51.28	47.22	0.788	41.00	39.70	0.975
	Faecal incontinence	8	19	0.860	20.40	13.88	0.133	28.20	33.33	0.688	33.30	16.70	0.032*
	Sore skin	14	14	0.992	14.96	7.40	0.128	20.51	13.89	0.527	26.90	7.70	0.023*
	Embarrassment	9	11	0.695	10.20	8.33	0.318	NR	NR	NR	38.50	28.20	0.180
	Impotence	49	53	0.154	50.45 (37)	48.33 (20)	0.767	51.85 (12)	66.67 (10)	0.472	41.0 (13)	51.0 (17)	0.483
	Dyspareunia	7	10	< 0.001*	0 (12)	2.08 (16)	0.802	8.33 (4)	13.33 (5)	0.761	7.4 (9)	8.3 (5)	0.905

**Table 4:** EORTC QLQ-C30

EORTC QLQ-C30		Kyong Ha et al. [45] (2021)			Bjoern et al. [41] (2019)			Mora et al. [47] (2018)			Veltcamp Helbach et al. [42] (2018)		
		TaTME	LaTME	p	TaTME	LaTME	p	TaTME	LaTME	p	TaTME	LaTME	p
Global health status		66.67	66.67	0.456	77.72	79.86	0.625	73.96	72.62	0.874	79.60	83.60	0.208
Functional scales	Physical	100	100	0.937	88.29	89.81	0.688	92.50	86.67	0.273	83.20	88.10	0.128
	Role	100	100	0.280	84.69	85.18	0.772	91.67	79.76	0.255	80.20	89.50	0.042*
	Emotional	100	100	0.368	87.07	93.51	0.041*	89.58	77.38	0.031*	89.40	90.10	0.887
	Cognitive	100	100	0.304	90.47	95.83	0.069	85.42	83.33	0.775	89.40	90.10	0.860
	Social	100	100	0.464	88.43	93.51	0.272	91.67	86.90	0.604	87.70	92.60	0.093
Symptom scales	Fatigue	0	0	0.684	48.63	44.44	0.392	15.97	22.61	0.462	26.50	14.00	0.021*
	N&V	0	0	0.357	2.04	1.38	0.978	1.04	0.00	0.359	3.10	2.50	0.987
	Pain	0	0	0.491	10.20	8.79	0.645	5.20	13.09	0.235	12.8	3.70	0.051
	Dyspnoea	0	0	0.489	12.24	4.62	0.063	16.67	14.28	0.814	23.50	9.90	0.214
	Insomnia	0	0	0.300	18.36	14.81	0.449	14.58	21.42	0.426	18	14.80	0.385
	Appetite Loss	0	0	0.295	10.88	2.77	0.052	12.50	2.38	0.190	7.40	2.50	0.358
	Constipation	0	0	0.491	10.88	6.48	0.549	22.92	33.33	0.381	8.60	9.90	0.763
	Diarrhoea	0	0	0.861	17.68	4.62	0.009*	14.60	23.80	0.372	16	3.70	0.070
	Financial difficulties	0	0	0.286	1.36	0.00	0.223	NR	NR	NR	14.80	2.40	0.032*

### Functional outcomes

#### LARS

**Fig. 2:**
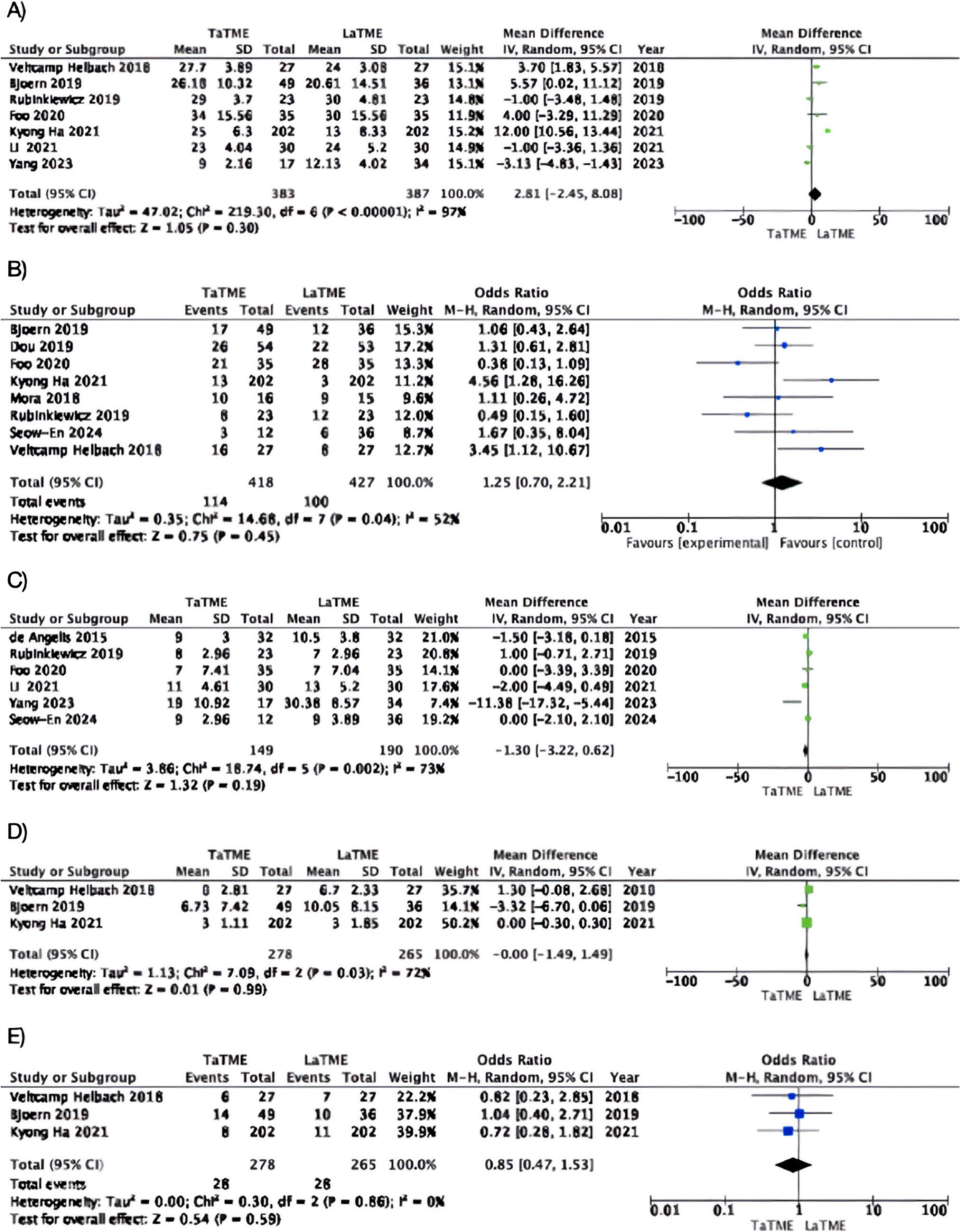
Forest plots of mean differences of (**a**) low anterior resection syndrome (LARS) score, (**b**) low anterior resection syndrome (LARS) major events, (**c**) Wexner score, (**d**) International Prostate Syndrome Core (IPSS), (**e**) International Prostate Syndrome Core (IPSS) comparison in case of moderate and severe symptoms; TaTME transanal total mesorectal excision, LaTME laparoscopic total mesorectal excision

Seven studies [41, 42, 45, 46, 48–50] (taTME group *n* = 421; LaTME group *n* = 421) examined LARS scores (Table 1s), revealing a mean score of 26.24 ± 5.32 in the taTME group and 23.84 ± 25.53 in the LaTME group. No statistically significant distinction emerged between the two groups, although the mean difference (MD) favoured the taTME group (MD: 2.81, 95% CI: − 2.45 – 8.08, *p*= 0.3; I2 = 97%) (Fig. 2a).

The present analysis shows substantial heterogeneity (I^2^ = 97%). After removing two studies [42, 45], heterogeneity decreased significantly to 67%. This suggests that these studies were major contributors to the overall variability. Significantly, the overall results of the meta-analysis did not change after their removal (MD: -0.43, 95% CI: − 2.81 – 1.96, *p* = 0.73; I2 = 67%).

Overall, 27.27% of taTME patients and 23.42% of LaTME patients reported major LARS. In this case, as well, the analysis of the population with major LARS does not show statistical significance between taTME and LaTME groups (OR: 1.25, CI: 0.7 – 2.21, *p* = 0.45; I^2^ = 54%).

#### Jorge-Wexner scale

Six studies [19, 44, 46, 48–50] (taTME group *n* = 149; LaTME group *n* = 190) assessed the severity of fecal incontinence using the Jorge-Wexner score [33] (Table 1s). While the average score was slightly lower for the taTME group (10.29) compared to the LaTME group (12.41), this difference was not statistically significant (MD: -1.3, 95% CI: -3.22 to 0.62, *p* = 0.19) (Fig. 2b). Moderate inconsistency in results across studies (I^2^ = 73%) was noted.

#### IPSS

Three studies [41, 42, 45] (taTME group *n* = 278; LaTME group *n* = 265) provided data on IPSS [32] (Table 2s) in patients undergoing taTME and LaTME. The IPSS score is 5.8 ± 2.67 in the taTME group and 5.81 ± 3.34 in the LaTME group. Statistical analysis revealed no significant differences between the two groups, with the mean difference (MD) favouring the taTME group (MD: 0.0, 95% CI: − 1.49 – 1.49, *p* = 0.99; I^2^ = 72%) (Fig. 2d).

A total of 28 (10.07%) patients in the taTME group and 28 (10.56%) patients in the LaTME group exhibited moderate or severe IPSS symptoms. Statistical analysis between these subgroups revealed no significant disparities between the two groups, with the mean difference (MD) favouring the taTME group (MD: 0.85, 95% CI: 0.47–1.53, *p* = 0.52; I^2^ = 0%) (Fig. 2e).

#### EORTC QLQ-C29

Four studies [41, 42, 47, 50] (taTME group *n* = 122; LaTME group *n* = 108) reported the QLQ-C29 questionnaire results [34] (Table 3). The assessment revealed that sexual interest (women), dyspareunia, buttock pain, altered taste, hair loss, fecal incontinence, and sore skin were significantly more prevalent in the taTME group (*p* = 0.039, < 0.001, 0.011, 0.047, 0.010, 0.032, and 0.023, respectively). In contrast, abdominal pain and bloating were significantly more frequent in the LaTME group (*p* = 0.044 and 0.042, respectively).

#### EORTC QLQ-C30

Four studies [41, 42, 45, 47] (taTME group *n* = 294; LaTME group *n* = 280) reported the QLQ-C30 questionnaire [35, 36] results (Table 4). The questionnaire indicated that diarrhea, fatigue, and financial difficulties were significantly more common in the LaTME group (*p* = 0.009, 0.021, and 0.032). Additionally, role functioning improved considerably in the LaTME group (*p* = 0.042). Emotional function yielded conflicting significant results in two studies, with Bjoern et al. [41] favouring LaTME (*p* = 0.041) and Mora et al. [47] favouring taTME (*p* = 0.031). Across all studies, we noted no statistically significant differences in global health status scores.

## Discussion

Quality of life and functional outcomes have been recognized as crucial outcome measures after TME surgery, alongside traditional oncological endpoints. This meta-analysis aimed to compare the functional outcomes and QoL between patients undergoing LaTME and taTME. The results indicate that the two techniques provide similar overall functional outcomes, with no statistically significant differences across various scoring systems and QoL questionnaires. Similar conclusions have been reached by Choy KT et al., who reported comparable functional outcomes with both surgical techniques, including LARS, incontinence scores, and QoL [23]. Transanal TME has recently emerged as an effective technique for treating tumors located in the lower rectum. This approach involves a bottom-up dissection starting transanally, which allows for precise establishment of the distal margin and facilitates dissection in anatomically challenging areas such as a narrow pelvis or patients with obesity.

Although the technique is associated with favorable short-term oncological outcomes and low conversion rates to open surgery [51], concerns have been rising regarding poor-postoperative functional outcomes due to the low anastomosis and the potential damage to the anal sphincter complex caused by the sustained dilation required during the procedure. Studies have shown that both taTME and LaTME result in decreased anal sphincter pressures postoperatively, with significant reductions observed in squeeze pressures [24, 25]. However, these changes do not appear to differ significantly between the taTME and LaTME approaches, suggesting that the transanal approach does not inherently confer a higher risk of sphincter damage than the laparoscopic approach [23, 24].

Furthermore, the long-term follow-up studies indicate that the initial postoperative deterioration in sphincter function may improve over time, with no significant differences in anorectal manometry outcomes between taTME and LaTME after extended periods [24, 25].

Using validated questionnaires, a phase II North American multicenter prospective observational trial assessed 100 patients after taTME for rectal cancer. The study revealed that defecatory function and fecal continence initially worsened post-ileostomy closure but showed significant improvement by 12–18 months, although they did not return to preoperative status. Urinary function remained stable, while sexual function declined and did not improve by 18 months post-taTME [52].

The results of this study showed that the LARS score did not significantly differ between taTME and LaTME. However, the taTME group had a slightly higher mean LARS score, indicating more severe bowel dysfunction, though this difference was not statistically significant. This suggests that while taTME may offer surgical advantages, it does not necessarily result in better bowel functional outcomes than LaTME. The high heterogeneity (I^2^ = 97%) among studies indicates variability in results, making it difficult to draw definitive conclusions. The high heterogeneity across studies can be attributed to variations in study design, differences in patient populations, variations in surgical techniques within the same approach (taTME or LaTME), the use of different questionnaires and scoring systems with inherent subjectivity, and varying lengths of follow-up. Similarly, the Jorge-Wexner scores did not differ significantly between groups, with taTME showing a slightly lower mean score, indicating less incontinence. However, this difference was still not statistically significant. Moderate inconsistency (I^2^ = 73%) suggests variability in findings across different studies investigating the same outcome measures, complicating the final interpretation of these findings.

Comparable results were also reported in studies that evaluated anorectal function using manometry in LaTME vs taTME patients. In particular, Bjoern & Perdawood report on similar mean resting pressure at anorectal manometry between taTME and LaTME (36.44 mmHg ± 18.514 vs. 36.58 mmHg ± 13.318, respectively, *p* = 0.981). Squeeze pressures were also comparable between the groups (125.00 mmHg ± 66.141 vs. 111.83 mmHg ± 51.111, respectively, *p* = 0.533). These findings suggest that the internal sphincter function is similarly impaired following both surgical techniques, while the external sphincter function remains within normal ranges [24]. De Simone et al. also evaluated anorectal function and QoL of 33 patients who underwent taTME surgery for mid- or low rectal cancer and completed a 12-month follow-up using questionnaires, anorectal manometry, and 3D endoanal ultrasonography (3D-EAUS). All the evaluations were performed before and after surgery, allowing for a homogenous comparison. At manometry, results showed a statistically significant decrease in mean resting pressure at the 12-month follow-up (from 40.7 mmHg to 32.2 mmHg, *p* = 0.012). However, maximum resting pressure and maximum squeeze pressure did not change significantly. At the 3D-EAUS, 15% of patients showed increased inhomogeneity of the sphincter fibers, which could indicate some degree of muscle damage or alteration [53].

In terms of sexual function, limited data exists, but the available studies indicate no significant differences between the two techniques. Sexual dysfunction following taTME for rectal cancer is a recognized complication with varying impacts on erectile and ejaculatory functions. Studies indicate that sexual dysfunction, including reduced erectile function and ejaculatory problems, is common postoperatively. Interestingly, Nishizawa Y et al. reported significant erectile dysfunction in 80% of men at three months postoperatively, which slightly improved to 76% at 12 months [54]. Another study highlighted that sexual impairment after taTME remains a serious concern, with nearly half of the patients experiencing impaired spontaneous erectile function [55].

Conversely, Pontallier A. et al. demonstrated a better erectile function with a significantly higher rate of sexual activity in the transanal group compared to the conventional laparoscopic approach (71% vs 39%, *p* = 0.02) [56].

In our study, the EORTC QLQ-C29 demonstrated that a low sex drive in women and dyspareunia were significantly more prevalent in the taTME group. Conversely, in men, sexual interest and potency were preserved. Regarding urogenital function, there is no clear evidence that taTME results in more dysfunction than LaTME. Both techniques are associated with similar IPSS scores, suggesting comparable impact on urogenital function. In our analysis, both groups had similar mean IPSS scores, and the distribution of moderate or severe symptoms was comparable, indicating that neither surgical technique has a clear advantage in preserving urogenital function. However, the high variability and subjective nature of these assessments warrant caution in interpretation. Similar conclusions have been reached by Bjoern et al. using the International Consultation on Incontinence Questionnaire-Male/Female Lower Urinary Tract Symptoms (ICIQ-MLUTS/FLUTS). No significant differences in urinary function or bother scores between baseline and follow-ups were found. However, a trend towards increased urinary incontinence and total bother scores was observed in male patients at the second follow-up at 13.5 months (*p* = 0.060 and *p*= 0.052, respectively) [24].

Our meta-analysis has several limitations. First, the high heterogeneity of the included studies, as indicated by the variability in the LARS and Jorge-Wexner scores, prevents drawing definitive conclusions. This variability may originate from differences in study designs, patient populations, distance of the tumor from anal verge, neo- and adjuvant regimens, variations in surgical techniques, in the timeline of administered questionnaires to assess patient functional status after surgery, and perioperative care protocols. Second, the reliance on subjective scoring systems such as LARS, Jorge-Wexner, and IPSS introduces the potential for bias and variability in patient self-reporting, which may overestimate or underestimate the true impact on functional outcomes and quality of life. Additionally, the lack of long-term follow-up data limits our understanding of the prolonged effects of taTME and LaTME on urogenital and sexual function. Finally, the limited number of studies specifically assessing sexual function further limits the comprehensiveness of our findings in this important aspect of patient well-being. Future research should address these limitations by incorporating more objective measures, like pre- and post-operative anorectal manometry and endoanal ultrasound, ensuring consistent methodologies, and extending the follow-up period to capture long-term outcomes.

## Conclusions

Functional outcomes and QoL are similar for rectal cancer patients who underwent either taTME or LaTME. However, the evidence is limited by the heterogeneity of studies and the reliance on subjective outcome measures. Further research is needed to confirm these findings and better understand the long-term impact of the possible functional sequelae of these surgical approaches.

## Supplementary Information

Below is the link to the electronic supplementary material.

Supplementary file1 (DOCX 11 KB)

## Data Availability

No datasets were generated or analysed during the current study.
