# Supplementary material for: Quality of life and functional outcomes after laparoscopic total mesorectal excision (LaTME) and transanal total mesorectal excision (taTME) for rectal cancer. an updated meta-analysis
Source: Int J Colorectal Dis. 2024 Aug 9;39(1):129. doi: 10.1007/s00384-024-04703-x (PMC11315702; doi:10.1007/s00384-024-04703-x)
Supplement: Supplementary file 1 — Supplementary file1 (DOCX 11 KB) [file 384_2024_4703_MOESM1_ESM.docx]

**Table 1s: LARS and Jorge-Wexner scale**

| **Author and year** | **LARS** | | | | | | | | **Jorge-Wexner scale** | |
| --- | --- | --- | --- | --- | --- | --- | --- | --- | --- | --- |
|  | Category | | | | | | LARS Score | | median (range) | |
|  | TaTME | | | LaTME | | | TaTME | LaTME | TaTME | LaTME |
|  | None | Minor | Major | None | Minor | Major | mean (SD) | mean (SD) |  | |
| Seow-En et al. [^45^](https://www.zotero.org/google-docs/?guliY2), 2024 | 7 | 2 | 3 | 19 | 9 | 6 | NR | NR | 9 ± 2.96 | 9 ± 3.89 |
| Yang et al. [^51^](https://www.zotero.org/google-docs/?9recVx), 2023 | NR | NR | NR | NR | NR | NR | 9.00 ± 2.16 | 12.13 ± 4.02 | 19.00 ± 10.92 | 30.38 ± 8.57 |
| Li et al. [^52^](https://www.zotero.org/google-docs/?YuE7un), 2021 | 8 | 7 | 15 | 10 | 7 | 13 | 23 ± 4.04 | 24 ± 5.20 | 11 ± 4.61 | 13 ± 5.20 |
| Kyong Ha et al. [^47^](https://www.zotero.org/google-docs/?ce7YNQ), 2021 | 14 | 15 | 13 | 28 | 13 | 3 | 25 ± 6.3 | 13 ± 8.33 | NR | NR |
| Foo et al. [^48^](https://www.zotero.org/google-docs/?5Iex80), 2020 | 8 | 6 | 21 | 12 | 5 | 18 | 34 ± 15.56 | 30 ± 15.56 | 7 ± 7.41 | 7 ± 7.04 |
| Bjoern et al. [^42^](https://www.zotero.org/google-docs/?AUI2kQ), 2019 | 17 | 15 | 17 | 16 | 8 | 12 | 26.18 ± 10.32 | 20.61 ± 14.51 | NR | NR |
| Rubinkiewicz et al. [^50^](https://www.zotero.org/google-docs/?I0XwQr), 2019 | 3 | 12 | 8 | 2 | 9 | 12 | 29 ± 3.7 | 30 ± 4.81 | 8 ± 2.96 | 7 ± 2.96 |
| Dou et al. [^44^](https://www.zotero.org/google-docs/?C1UFxn), 2019 | 11 | 17 | 26 | 16 | 15 | 22 | NR | NR | NR | NR |
| Mora et al. [^49^](https://www.zotero.org/google-docs/?J51uG9), 2018 | 3 | 3 | 10 | 4 | 2 | 9 | NR | NR | NR | NR |
| Veltcamp Helbach et al. [^43^](https://www.zotero.org/google-docs/?pPW1d8), 2018 | 7 | 4 | 16 | 11 | 8 | 8 | 27.7 ± 3.89 | 24 ± 3.08 | NR | NR |
| de' Angelis et al. [^19^](https://www.zotero.org/google-docs/?Fd2tOt), 2015 | NR | NR | NR | NR | NR | NR | NR | NR | 9 ± 3 | 10.5 ± 3.8 |

**NR = not reported**

**Table 2s: IPSS**

| **Author and year** | **IPSS** | | | | | | | | | |
| --- | --- | --- | --- | --- | --- | --- | --- | --- | --- | --- |
|  | Symptoms (%) | | | | | | | | IPSS Score | |
|  | TaTME | | | | LaTME | | | | TaTME | LaTME |
|  | None | Mild | Moderate | Severe | None | Mild | Moderate | Severe | mean (SD) | mean (SD) |
| Kyong Ha et al.[^47^](https://www.zotero.org/google-docs/?w72pla), 2021 | NR | 45 (84.9) | 7 (13.2) | 1 (1.9) | NR | 51 (82.3) | 11 (17.7) | 0 | 3 (1.11) | 3 (1.85) |
| Bjoern et al. [^42^](https://www.zotero.org/google-docs/?EBuhs6), 2019 | 6 (16.2) | 17 (45.9) | 12 (32.4) | 2 (5.4) | 1 (5) | 9 (45) | 8 (40) | 2 (10) | 6.73 (7.42) | 10.05 (8.15) |
| Veltcamp Helbach et al. [^43^](https://www.zotero.org/google-docs/?zKMXcR), 2018 | NR | 12 (66.7) | 5 (27.8) | 1 (5.6) | NR | 7 (50) | 7 (50) | 0 | 8 (2.81) | 6.7 (2.33) |
